# Endoscopic Management of Drain Inclusion in the Gastric Pouch after Gastrojejunal Leakage after Laparoscopic Roux-en-Y Gastric Bypass for the Treatment of Morbid Obesity (LRYGBP)

**DOI:** 10.1155/2010/891345

**Published:** 2010-06-20

**Authors:** Ramon Vilallonga, José Manuel Fort, Oscar Gonzalez, Juan Antonio Baena, Albert Lecube, Josè Salord, Manel Armengol Carrasco, Josep Ramon Armengol-Miró

**Affiliations:** ^1^Endocrine and Metabolic Unit, General Surgery Department, Universitary Hospital Vall d'Hebron, Passeig de la Vall d'Hebron, 119-129, 08035 Barcelona, Spain; ^2^Endocrinology Department, Universitary Hospital Vall d'Hebron, 08035 Barcelona, Spain

## Abstract

*Background*. Drain inclusion inside the gastric pouch is rare and can represent an important source of morbidity and mortality associated with laparocopic Roux-en-Y gastric bypass (LRYGBP). These leaks can become chronic and challenging. Surgical options are often unsuccessful. We present the endoscopic management of four patients with drain inclusion. *Patients*. All four obese morbidly patients underwent LRYGBP and presented a gastro-jejunal fistula after acute anastomotic leakage. During follow-up endoscopy the drain was found inside the gastric pouch. It was moved into the abdominal cavity. Fistula debit reduced significantly and closed. *Results*. Gastric leak closure in less than 24 hours was achieved in all, with complete resolution of symptoms. These patients benefited exclusively from endoscopic treatment. *Conclusions*. Endoscopy is useful and technically feasible in chronic fistulas. This procedure is a less invasive alternative to traditional surgical revision. Other therapeutic strategies can be used such as clips and fibrin glue. Drains should not be placed in contact with the anastomosis or stapled lines. Drain inclusion must be suspected when fistula debit suddenly arises. If so, endoscopy is indicated for diagnostic accuracy. Under endoscopy vision, the drain is gently removed from the gastric reservoir leading to sudden and complete resolution of the fistula.

## 1. Introduction

Laparoscopic Roux-en-Y gastric bypass (LRYGBP) is one of the most frequently performed bariatric procedures worldwide and complications such as postoperative gastrocutaneous fistula (GCF) are infrequent and difficult to treat [1]. Leaks can occur in 0.5% to 4.4% of patients who undergo LRYGBP operations, resulting in significant morbidity with peritonitis, abscess formation, sepsis, multiorgan failure, and eventual death [2–5]. Early detection of leaks is necessary and is proven to reduce morbidity and mortality. Leaks may appear in the gastric remanent either in staple line or in the gastrojejunal anastomosis itself. Some surgeons feel that most leaks can be managed conservatively with total parenteral nutrition and broad-spectrum intravenous antibiotics as long as adequate drainage has been achieved. Operative intervention with large-drain placement and/or surgical repair is, however, necessary when sepsis and/or symptoms of sepsis developed.

Though, spontaneous closure of GCF occurs in 90% of cases within 2 weeks, but the mortality rate can reach 85% among patients presenting with sepsis [6]. Because of the surgical difficulty, however, successful primary surgical repair of the leak is difficult. This is the reason why an endoscopic approach can be useful and less aggressive in order to treat these complications.

We present herein four patients with gastrojejunal fistula with drain inclusion into the gastric pouch after LRYGBP. 

### 1.1. Patient 1

A 51-year-old man with BMI = 49,5 Kg/m^2^ hypertension underwent a Roux-en-Y gastric bypass. Extensive adhesions were noted during the surgery. The routine gastrografin (Bristol-Myers Squibb, Princeton, NJ) swallow studies performed at the fourth postoperative day showed a leak at the gastrojejunal anastomosis. Though the patient was completely asymptomatic, he underwent surgery and no anastomotic leakage was found in the jejunojejunal anastomosis. A Jackson-Pratt drain was placed next to the previous gastrojejunal anastomosis. During the postoperative period, the patient was treated with IV antibiotics and on the 7th day after surgery, drainage showed the presence of intestinal fluid. A gastrografin swallow demonstrated a leak from the proximal gastric pouch to the peritoneum that was not seen in the reoperation. Patient needed ambulatory parenteral nutrition with the fistula debit always inferior to 30 cc/24 hours. Patient was discharged after 20 days. After two months the fistula debit suddenly arised up to 300 cc/24 hours.

Therefore, the patient underwent ambulatory endoscopy exploration. On the endoscopy, 10 cm of the tip of the drain tube was visible inside the gastric pouch, just above the gastrojejunal anastomosis. The drain was pushed gently into the abdominal cavity, until complete disappearance from the pouch. There were no complications associated with the procedure. No clips were used to close the 6 mm anastomotic defect. Fistula debit decreased immediately to 0 cc. One week later, the drain was removed and the patient was discharged from the hospital with regular intake.

### 1.2. Patient 2

A 63-year-old female with BMI = 43 Kg/m^2^ underwent an LRYGBP. Ten days after the operation, she presented a positive methylene blue swallow test, with methylene blue through the drain. She was treated with IV antibiotics with no systemic worsening. After three weeks, a gastrostomy was performed and the patient began enteral intake through the gastrostomy. Patient was discharged with the drain placed after 25 days. After two months, she presented with an epigastric pain and an endoscopy showed an acute ulcer due to eroding effect of the drain that was inside the gastric pouch. Drainage was moved into the abdominal cavity as in the other patient. Fistula debit decreased immediately and closed spontaneously.

### 1.3. Patient 3

A 54-year-old man with BMI = 49 Kg/m^2^ and hypertension underwent an LRYGBP. He presented with fever and abdominal pain two days after surgery. Patient underwent urgent surgical exploration. The anastomotic leakage was sutured and a penrose drain was left next to the anastomosis. On the seventh postoperative day, a 30 cc fistula debit began. Because of a sudden increase (up to 250 cc/24 hours) of fistula debit observed after 3 weeks, an endoscopy was performed. The drain was also visible inside the gastric pouch. It was removed to the abdominal cavity under endoscopic vision as in the other cases and fistula debit decreased immediately so that drainage could be finally removed. Patient was discharged after 36 days.

### 1.4. Patient 4

**Figure 1:**
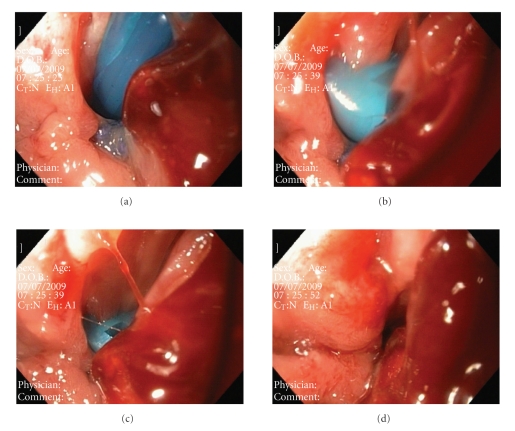
We show in these four sequences of images the drain inside the gastric pouch and how it has been pushed to the abdominal cavity by pulling it from the drain tube outsider the abdomen.

A 50-year-old woman with BMI = 40 Kg/m^2^, DM-2 and hypertension underwent an LRYGBP. She presented with an acute digestive haemorrhage and abdominal pain two days after surgery. She required blood and plasma transfusions and antithrombotic drugs suspension. After 5 days of operation, the patient began with suppuration from the drain and a methylene blue test confirmed the presence of a fistula, with a 20 cc/24 hours debit. At the twenty-seventh day after surgery, an endoscopy was performed. The drain was also visible inside the gastric pouch (Figure 1). It was removed to the abdominal cavity under endoscopic vision as in the other cases and fistula debit decreased immediately. Three days after, a gastrografin (Bristol-Myers Squibb, Princeton, NJ) swallow confirmed the closure of the fistula and the patient began the oral intake. Patient was ten discharged after 32 days.

## 2. Discussion

Laparoscopic bariatric operations have become standard surgical treatment for morbid obesity. LRYGBP is a technically demanding operation and technical errors can cause serious complications unless a learning curve is correctly observed [7]. One of the most serious related mortality complications is anastomotic dehiscence and leak [2, 3, 8, 9]. Gastric pouch leaks occur in up to 5.6% of LRYGBP operations, resulting in significant morbidity, sepsis, multiorgan failure, and eventual death [2–5, 10, 11]. 

It is crucial to detect early any kind of leak in order to reduce morbidity and mortality. Detecting postoperative leaks can be challenging. Clinical signs such as fever, tachycardia and abdominal pain may alert the clinician. The role of drainage in the early diagnosis of fistula is well recognised.

Assessment of peritonitis in these patients can be difficult owing to obesity and few findings from physical examination, and we consider that a CT scan should be performed. The postoperative period is also a problem when some patients are discharged two or three days after surgery. The postoperative observation period is short and some authors have showed the convenience of leaving a drain even after patient discharge [3]. Another aspect is the rapid introduction of the oral intake that may lead to more severe peritonitis, which will increase the morbimortality of the patient [2]. This last argument is important in the context of leak detection because, despite aggressive medical and/or surgical management, some patients will develop ongoing sepsis, multisystem organ failure, and death. 

The surgical technique is a factor to take into consideration. Some authors have reported the incidence rates of leak in vertical gastroplasty (whether banded [12] or modified [13]), in which disruption of the staple lines (vertical or circular) occurs in about 2.0% to 4.5% of cases [14, 15].

We routinely perform intraoperative testing, infusing methylene blue through the orogastric tube under laparoscopic guidance in order to minimise postoperative leaks. This measure has already been recommended by other authors [16]. Additionally, an intraoperative endoscopy with low-pressure air insufflation can be performed to evaluate the gastrojejunostomy [2, 8].

Management of these patients is challenging. Surgical options for treating chronic gastric leaks have a very high operative risk because of an altered anatomy with chronic inflammation and also because of the multiple adhesions that will be present. This is why a patient who presents with an anastomotic leak without major systemic dehydration, electrolyte derangement, malnutrition, infection, sepsis, multiple organ dysfunction or failure is sometimes treated conservatively, as was Patient 2. This treatment must include a proper restoration of blood volume, correction of electrolyte imbalance, control of infection and sepsis, alimentary tract rest, elimination of any downstream resistance, and optimal nutrition [1, 14, 17, 18]. Fistula control must be performed with an adequate drain placed next to the anastomosis and the use of total parenteral nutrition, in combination with administration of somatostatin or its analogue octreotide, is mandatory [3]. Surgical treatment with repair of the leak must be performed when the patient is at risk of dying and surgery becomes life-saving. Because of the technical difficulty, however, the inability to close the tissue defect can lead to the development of a new fistula [15]. However, these patients are in some way managed in the standard protocol, but because of a sudden increase of the drain, a complication related to the drain should be confirmed. All patients, should undergo standard management according to protocols [19].

Of those who survive the initial sepsis, if it appears, some develop persistent leaks, despite adequate drainage of associated fluid collections, as in our patients. The use of drains allows early identification of leaks if gastrointestinal effluent is noted. The drain can also reduce the possibility of collection formation, and their low output is the reason why most of the postoperative leaks from the gastrojejunostomy generally resolve spontaneously. We have found, however, one reference to the drain inclusion in the gastric pouch after surgery or resurgery with endoscopically treatment [20]. In most cases, closed drainage systems can adequately handle these low volumes of saliva and gastric content without the need for reoperation but this has not been the case for our patients who had to undergo an endoscopy to check the anastomotic leak and reveal that the drain was inside the pouch [8]. Of course, all the drain complications were following a “first-time” leak and it could be that an actual leak complication may predispose the subsequent complication of drain inclusion into the pouch.

Once the patient presents with a controlled fistula some therapeutic options have been described if closure is not spontaneous. Endoscopy should be performed after initial recovery of the patient and especially if debit is important, in order to rule out, the possibility of drain inclusion. However, in the fourth patient, daily debit was only of 20 cc. Much interesting information concerning the leak can be given. We have not found any report of endoscopic gastric leak repair after LRYGBP, while there are analogous reports of oesophageal fistula repair. Both acute oesophageal perforations and chronic oesophageal fistulas have been treated with endoclips [20–22]. Another conservative method is the use of fibrin glue endoscopically placed to close an enteric fistula [20, 23–26]. Fibrin tissue glue has been used for more than ten years in different fields of surgery [27–29] and in morbidly obese patients who have undergone vertical gastroplasty with good results [30]. We might consider the possibility of using low aspirative pressure drains, and these should be placed close to the anastomosis. In our four patients we did not use any fibrin adhesive because the simple mobilisation of the drain permitted the immediate collapse and closure of the defect. In some cases reported, multiple applications and relatively large volumes of fibrin adhesive are required to close the pouch defect [31]. Some authors have proven the utility of the fibrin glue, which promotes neovascularisation and fibroblast proliferation [31–33].

In a more recent report, Gumbbs treated two patients of fourteen (14.29%) who underwent vertical gastroplasty and developed a nonhealing gastrocutaneous fistula with endoscopic application of a fibrin sealant under direct vision. This procedure was described as simple, safe, and effective and, in some cases, life-saving [34].

In addition to clips and glue, argon plasma coagulation and self-expanding covered metal stents have been used to close leaks and perforations by acting as a fluid barrier [35]. Some potential complications have been described, such as stent migration and tissue overgrowth. Treating complications like stent removal can also be very challenging.

Even if our patients were treated endoscopically, none of the endoscopic techniques described was used. Endoscopy showed the drain inclusion, however, and allowed us to control the manoeuvre to push the drain into the abdominal cavity. A more common way to manage such drains is to withdraw the drains 3-4 cm on a weekly basis, turning the leak back to a controlled fistula tract. This may have avoided the need to push back the drain endoscopically. Clip placement, glueing or luminal stent placement might be considered on another occasion. Also some authors have reviewed the endoscopic management of megaobese patients with BMI over 70 Kg/m^2^. In these patients, Suture-line reinforcement, at least selectively in the middle-upper portion of the staple line and in super-super-obese patients, is recommended to decrease the incidence of specific complications such as fistula [36]. Other authors have performed a laparoscopic fistulotomy with debridement [37]. 

## 3. Conclusions

Patients who present with a chronic leak must undergo an endoscopy to avoid the possibility of drain inclusion in the gastric pouch. In these cases, endoscopic repair may be a safe and effective option for patients who would otherwise undergo complex surgical revision.
